# Molecular detection of *Mycobacterium ulcerans* in the environment and its relationship with Buruli ulcer occurrence in Zio and Yoto districts of maritime region in Togo

**DOI:** 10.1371/journal.pntd.0006455

**Published:** 2018-05-21

**Authors:** Issaka Maman, Tchadjobo Tchacondo, Abiba Banla Kere, Marcus Beissner, Kossi Badziklou, Ekanao Tedihou, Edith Nyaku, Komi Amekuse, Franz Xaver Wiedemann, Damintoti Simplice Karou, Gisela Bretzel

**Affiliations:** 1 National reference laboratory for Buruli ulcer disease in Togo, Institut national d’hygiène (INH), Lomé, Togo; 2 Laboratoire des sciences biomédicales et substances bioactives (LSBSB), Ecole supérieure des techniques biologiques et alimentaires (ESTBA), Université de Lomé, Lomé, Togo; 3 Department for infectious diseases and tropical medicine (DITM), Medical center of the University of Munich (LMU), Munich, Germany; 4 Laboratoire de défense des cultures -Laboratoire national de biosécurité, Institut togolais de recherche agronomique, ITRA, Lomé, Togo; 5 German leprosy and tuberculosis relief association (DAHW-T), Togo office, Lomé, Togo; Hospital Infantil de Mexico Federico Gomez, UNITED STATES

## Abstract

**Background:**

Buruli Ulcer (BU) is a neglected tropical skin infection caused by *Mycobacterium ulcerans*. Residence near aquatic areas has been identified as an important source of transmission of *M*. *ulcerans* with increased risk of contracting Buruli ulcer. However, the reservoir and the mode of transmission are not yet well known. The aim of this study was to identify the presence of *M*. *ulcerans* in the environment and its relationship with Buruli ulcer occurrence in Zio and Yoto districts of the maritime region in south Togo.

**Methods:**

A total of 219 environmental samples including soil (n = 119), water (n = 65), biofilms/plants (n = 29) and animals’ feces (n = 6) were collected in 17 villages of Zio and Yoto districts of the maritime region in Togo. DNA of *M*. *ulcerans* including *IS2404* and *IS2606* insertions sequences and mycolactone ketoreductase-B gene (KR-B) was detected using real time PCR amplification (qPCR) technique. In parallel, clinical samples of patients were tested to establish a comparison of the genetic profile of *M*. *ulcerans* between the two types of samples. A calibration curve was generated for *IS2404* from a synthetic gene of *M*. *ulcerans* Transposase pMUM001, the plasmid of virulence.

**Results:**

In the absence of inhibition of the qPCR, 6/219 (2.7%) samples were tested positive for *M*. *ulcerans* DNA containing three sequences (*IS2404/IS2606/*KR-B). Positive samples of *M*. *ulcerans* were consisting of biofilms/plants (3/29; 10.3%), water (1/65; 1.7%) and soil (2/119; 1.5%). Comparative analysis between DNA detected in environmental and clinical samples from BU patients showed the same genetic profile of *M*. *ulcerans* in the same environment. All these samples were collected in the environment of Haho and Zio rivers in the maritime region.

**Conclusion:**

This study confirms the presence of *M*. *ulcerans* in the environment of the Zio and Yoto districts of the maritime region of Togo. This may explain partially, the high rates of Buruli ulcer patients in this region. Also, water, plants and soil along the rivers could be possible reservoirs of the bacterium. Therefore, Haho and Zio rivers could be potential sources of infection with *M*. *ulcerans* in humans in these districts.

## Author summary

Buruli ulcer is a skin disease caused by *Mycobacterium ulcerans*. Although residence near aquatic areas has been identified as an important source of increased risk of contracting Buruli ulcer, the reservoir and the mode of transmission are not well known. To improve the understanding of the mode of transmission of *M*. *ulcerans* in human, we report here the first study of detection of *M*. *ulcerans* in the environment and its relationship with BU occurrence in Togo. This study confirms the presence of *M*. *ulcerans* DNA in the environment of Zio and Yoto districts of the maritime region. This study provides information on some possible reservoirs of the bacterium such as water, plants/biofilms and soil. Also, in this article we showed that the rivers of Haho and Zio could be potential sources of infection of *M*. *ulcerans* in human. Finally, data obtained from this study could explain partially the high rate of Buruli ulcer cases in these two districts of the maritime region in south Togo.

## Introduction

Buruli ulcer (BU) is an infectious skin disease caused by the *Mycobacterium ulcerans* [1–3]. BU is the third most common mycobacterial disease after tuberculosis and leprosy in immunocompetent hosts. Although the rate of mortality of Buruli ulcer is low, the serious morbidity caused by the disease includes functional disabilities that may result in permanent social, economic and developmental problems. At least 50% of those affected by BU are children aged less than 15 years. Rates of infections among males and females are equivalent [1–4]. Infection with *M*. *ulcerans* often leads to extensive destruction of skin and soft tissue with the formation of large ulcers, commonly on limbs [4]. Necrosis and ulceration are induced by a diffuse cytotoxic macrolide lipid called mycolactone, which represents the key of the pathogenesis of the disease. Mycolactone is the product of three major complex enzymes called polyketide synthases which are coded by *mlsA1* (51Kb), *mlsA2* (7 Kb) and *mlsB* (42 Kb) genes. These genes are located on the plasmid of virulence of the mycobacterium known as pMUM001[5–6].

To date, BU cases have been reported in over 30 countries, particularly in tropical and subtropical climate regions but also in temperate climate zones such as Japan and southern Australia [1–4]. BU is a neglected tropical disease (NTD) with a poorly known global prevalence and mainly affects remote rural African communities [7]. According to the WHO (2016), from an estimated 7,000 BU cases reported annually worldwide and more than 4,000 cases occurred in Sub-Saharan Africa. The largest numbers of reported BU cases were from West African countries, particularly from Ivory Coast (about 2,000 cases annually), Benin and Ghana, each of which reported about 1,000 cases a year (WHO, 2016) [1–4].

All BU cases reported must be confirmed by laboratory techniques as recommended by WHO such as direct smears for detection of acid fast bacilli (AFB), in vitro culture and PCR amplification targeting IS*2404* sequence [8–9]. However, given the fact that *M*. *ulcerans* is a slow-growing bacterium, it may take 8 to 12 weeks to confirm a case by culture and this could delay the implementation of the treatment [8–9]. The development of the conventional PCR technique targeting IS*2404* sequence, an insertion sequence present in more than 200 copies per *M*. *ulcerans* genome is therefore considered a more sensitive and faster technique to confirm BU cases [10–11]. This method has been also used for testing environmental samples and allowed to detect IS*2404* insertion, suggesting a probable presence of *M*. *ulcerans* in water samples [12–14], aquatic insects [15], plants [16] and fish [17]. Although this technique is highly sensitive and specific for *M*. *ulcerans* detection in clinical samples, its application on environmental samples remains difficult and non-specific due to the presence of PCR inhibitors and the existence of other environmental mycobacterial species harbouring *IS2404* such as *Mycobacterium lifandii*, *Mycobacterium pseudoshottsii and Mycobacterium marinum* [18–20]. In order to increase the level of specificity and reliability of PCR results as well as increasing testing speediness of clinical and environmental samples, Fyfe et al. [21] have developed two multiplex real time PCR (qPCR) targeting two insertion sequences (IS*2404* and IS*2606*) and Ketoreductase-B domain gene. This new method allowed to distinguish *M*. *ulcerans* from other Mycobacterium species that also contain IS*2404* sequence [21].

Several epidemiologic studies in Africa [12,21–23] and Australia [13–24] have identified aquatic sources as important sources of *M*. *ulcerans* transmission with a high risk of contracting Buruli ulcer. However, the exact mechanism of the transmission of the bacterium is still unknown. The absence of evidence for human-to-human transmission suggests that *M*. *ulcerans* is an environment microorganism [25]. Human-linked changes in the aquatic environment such as dam constructions on rivers, deforestation, agriculture and mining have led to environmental disturbance and may contribute to the spread of *M*. *ulcerans* [26–27]. This could increase the prevalence of Buruli ulcer cases in endemic areas and lead to the emergence of the bacterium in areas where the pathogen was previously absent [26].

In order to improve the understanding of the mode of transmission of *M*. *ulcerans*, it is important at a first stage, to determine the ecology of the bacterium. Thus, some studies used the method described by Fyfe et al. and have identified *M*. *ulcerans* reservoirs by detecting DNA in multiples environment samples [12–13,22,28,29].

In Togo, the first cases of Buruli ulcer have been described in 1996 by Portaels et al [30]. Since 2007, several collaborations with German leprosy and tuberculosis relief association in Togo (DAHWT) and the department of infectious and tropical medicine (DITM) of the University of Munich like the BuruliVac project between 2011 and 2013 have proved that Buruli ulcer is endemic in the maritime region in south Togo [31–32]. The availability of a national reference laboratory using PCR technique allows to confirm every year about 30 to 65 new cases of which 85% are from Zio and Yoto districts [32]. Some studies [31–37], mainly clinical were carried out in Togo on BU. However, no environmental data exist on this disease and the risk of infection in human in this country. The present study aims to determine the presence of *M*. *ulcerans* in the environment and its relationship with the Buruli ulcer occurrence in Zio and Yoto districts of the maritime region of Togo.

## Materials and methods

We conducted a cross-sectional study in two districts of maritime region in south Togo. The sample collection method was based on a non-standardised sampling. Then environmental samples were collected from May 19 to 30, 2015 in 17 villages of Zio and Yoto districts where more than 85% of confirmed BU patients originated.

### Sampling area

**Fig 1:**
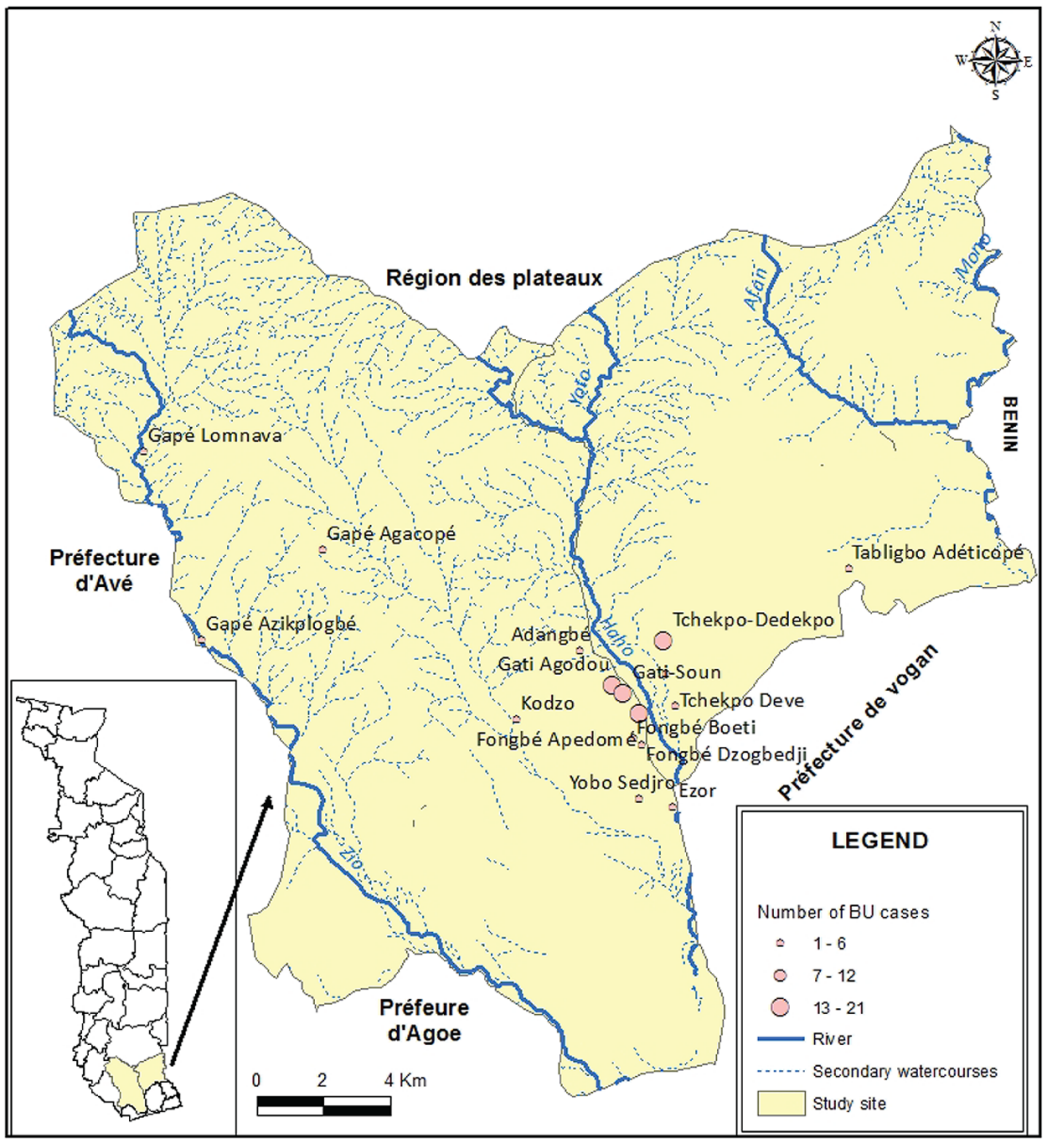
Maritime region map presenting villages surveyed, distribution of BU cases and hydrographic network. The circles in red correspond to the number of Buruli ulcer cases and placed at the 17 villages location in Districts of Zio and Yoto of the Maritime Region. Most of BU cases are located arround the watercourse of Haho with few cases observed near the Zio watercourses. These watercourses are main sources of activities with water contact that are associated with increasing risk of *M*. *ulcerans* infection.

The sampling sites are in maritime region of south Togo which covers the entire coastal part with an area of 6,359 km^2^. The population is estimated at 1,762,518 inhabitants in 2012. The climate is tropical and humid with two rainy seasons and two dry seasons. The region has a flat topography, with a low contrast characterized by a sedimentary basin that covers 4/5 of the region, a low altitude (50-80m on average) and crossed by the depression of the Lama. The soil, mainly clay remains soggy and muddy in the rainy season with stagnant water for several months. The hydrographic network comprises 3 large rivers which are on the one hand the Mono in the east and other hand, in the center, the Zio and the Haho. Both Zio and Haho have several small tributaries and flow into the “lac Togo” (Fig 1). All these streams have a low flow, closely linked to seasonal variations of precipitations [38].

### Environmental sampling

Samples were collected by three people consisting of two laboratory technicians and a health community volunteer (CHV). The volunteer has served as guide to find various collection sites in the villages. The samples consisted in water, plants, soil and animal feces.

### Water sample collection

Water samples were collected from ponds, open borehole, cisterns, pumps and borehole. At each point, 50 ml of water were taken and put in the Falcon tubes (greiner bio-one). In the rivers, samples were taken in the middle and at the edges upstream and downstream.

### Plant sample collection

Two to three most frequent plants or herbs (*Nymphea lotus*, *polygonome senegalensis*, *Ludwigia erecta*, *Pistia stratiotes*, *panicum maximum*) were collected from inside and along the edges upstream and downstream of the rivers. Each sample consisting of roots, stems and leaves was put in a same plastic sealable bag. To build up the biofilm, 50 ml of sterile water were added in the bag. Biofilms made were taken after 24 to 48 hours and put in 2 ml tube (Eppendorf).

### Soil samples collection

One gram of soil was taken at the surface and put in a 2 ml tube from houses as well as around of ponds, open cisterns, wells and pumps. For rivers, samples were taken along, upstream and downstream and at 5 m of the edge.

### Animal feces samples

Feces samples were taken from chicken, goats, sheep or cattle from house, henhouse and livestock farms.

All samples collected were stored in refrigerator at 4°C until laboratory analyses.

### Molecular analyses

The detection of *M*. *ulcerans* in environmental samples was performed using the following protocol (http://dx.doi.org/10.17504/protocols.io.pb7dirn).

### DNA extraction

Prior to DNA extraction, plant, soil and fecal samples were homogenized using the FastPrep-24 instrument (ver. 6004.2) at the laboratory of the Togolese agricultural research institute (ITRA, Lomé, Togo). Briefly, 200μl of each sample of soil, plant, and animals’ feces was transferred to a lysing matrix E tube in the presence of MT Buffer and sodium phosphate buffer After a rapid homogenization on the FastPrep-24 instrument for 40s at a speed setting at 6.0, the Matrix E tubes were directly centrifuged at 14000xg for 10 minutes to pellet debris.

For the biofilm and water samples, 200μl were transferred into the Matrix E tube and directly centrifuged at 14000xg for 10 minutes. After completion of the centrifugation, the supernatant was collected for DNA extraction.

The DNA was extracted at the molecular laboratory of the national institute of hygiene (INH, Lomé, Togo) using FastDNA Spin Kit for Soil for the 4 types of specimens following the recommendations of the manufacturer. One negative extraction control (sterile water) for each extraction batch has been added to check for a possible cross-contamination during the extraction process.

### PCR analysis

Real time PCR (qPCR) was performed as previously described by Fyfe et al. [21]. Three primers pairs with probes targeting sequences of two insertions sequences (*IS2404/IS2606*) and Ketereductase B-domain gene (KR-B) present on the *M*. *ulcerans* virulence plasmid pMUM001(GenBank accession no. BX649209) were used (Table 1). These targets were chosen because they were reported to be present in multiple copies in the *M*. *ulcerans* genome and are absent in the closely related species *Mycobacterium marinum* [19, 21].

**Table 1:** List of primers and probes sequences used for real time PCR (qPCR) targeting *IS2404* and *IS2606* insertions sequences and Ketoreductase-B domain gene, KR-B.

Primers and probes names^a^	Sequences (5'-3')	Nucleotides position^b^	Amplicons size
***IS2404* TF**	AAAGCACCACGCAGCATCT	27746–27762	**59**
***IS2404* TR**	AGCGACCCCAGTGGATTG	27787–27804	
***IS2404* TP**	**6 FAM**-CGTCCAACGCGATC-MGBNFQ	27768–27781	
***IS2606* TF**	CCGTCACAGACCAGGAAGAAG	28912–28932	**58**
***IS2606* TR**	TGCTGACGGAGTTGAAAAACC	28947–28969	
**IS*2606* TP**	**VIC**-TGTCGGCCACGCCG-MGBNFQ	28933–28946	
***KR* TF**	TCACGGCCTGCGATATCA	3178–3195	**65**
***KR* TR**	TTGTGTGGGCACTGAATTGAC	3222–3242	
***KR* TP**	**6 FAM**-ACCCCGAAGCACTG-MGBNFQ	3199–3212	

^a^TF, forward primer; TR: Reverse primer; TP: Probe.

^b^Nucleotide position based on the first copy of the amplicons in pMUM001 (GenBank accession no. BX649209).

To confirm the presence of *M*. *ulcerans* in an environmental sample, three consecutives qPCR runs were realized.

### First run: *IS2404*-qPCR

The first real time *IS2404*-qPCR run was quantitative using a Taqman probe targeting IS*2404* with three controls. An internal positive control (IPC,) to determine the level of inhibition, a no template control (NTC) and a positive control included in quadruplicate. A calibration curve was generated based on a serial dilution of known copies of *IS2404* from a synthetic gene of *M*. *ulcerans* Transposase pMUM001 (58 bp), the plasmid of virulence. All the dilutions of *IS2404*-DNA were tested both with samples to determine the sensitivity of the qPCR.

The amplification reaction was obtained from a mixture of 3μl of DNA extracted and 22μl of master mix which contained 0.5μl IPC DNA, 2.5μl IPC master mix, 1.25μl *IS2404* forward primer, 1.25μl *IS2404* reverse primer, 1.25 *IS2404* probe, 2.75μl water and 12.5 μl TaqMan Environmental Master mix 2.0. The reaction was run on ABI 7300 machine in the following conditions: 50°C for 2 minutes, 95°C for 15 minutes and 40 cycles of 95°C for 15 seconds and 60°C for 60 seconds.

All samples for which the internal control IPC and the *IS2404* insertion did not show the amplification curve were considered as inhibited samples. All inhibited samples were tested again after 10-fold dilution in DNase/RNase free water.

Samples found positive for *IS2404* have consecutively been tested in a semi-quantitative *IS2606*-qPCR run.

### Second run: *IS2606*-qPCR

The internal control IPC was no longer used, and the master mix prepared including 1.25μl of the *IS2606* forward primer, 1.25μl *IS2606* reverse primer, 1.25 μl *IS2606* probe, 5.75μl water and 12.5 μl du TaqMan Environmental Master mix 2.0. The amplification reaction was carried out from a mixture of 3μl DNA extract and 22μl master mix with the amplification conditions as described in the *IS2404*-PCR.

After the second run, all samples that were positive for both insertions sequences (*IS2404/IS2606*) were analyzed for detection of the mycolactone gene, KR-B.

### Third run: KR-qPCR

In this semi-quantitative qPCR, the KR gene was amplified from samples positive for the two insertions sequences (*IS2404 and IS2606*). The amplification reaction was composed of a mixture of 5μl DNA extract and 20μl of master mix in the same conditions as the *IS2606*-qPCR.

All samples were tested in duplicate. An environmental sample was considered positive for *M*. *ulcerans* if the qPCR was positive for the two insertions sequences (*IS2404/IS2606*) and KR-B gene (with a threshold cycle, Ct<40) in replicates and if the difference (ΔCt) between (*IS2404-IS2606*) was < 7.

To compare the genetic profile of the *M*. *ulcerans* strains detected in the clinical and environmental samples, 50 DNA extracts from clinical samples of BU patients were tested for the two insertions sequences (*IS2404/IS2606*) and KR-B gene in the same conditions of qPCR as for environmental samples. The clinical DNA was obtained from extraction of 31 liquid of fine needle aspiration (FNA) from nodule and 19 swabs collected from ulcers. The DNA extraction was performed with the Gentra Purgene DNA extraction kit as previously described [32].

### Statistical analysis

Statistical analysis was carried out by SPSS software (Statistical Package for Social Science, Version 16.0, SPSS Inc. and Chicago, IL). Student t-test was used for comparison of proportion of *IS2404* and *IS2606* insertions sequences and KR-B gene between different matrices with significant level set at p≤0.05.

### Ethics approval and consent to participate

The study protocol was approved by the National Program for Buruli Ulcer Control, (Authorization No.006/2014/MS/DGS/DSSP/PNLUB-LP) and the Ministry of Health as an integral part of the surveillance of the disease. However, this study did not require a review of the ethics committee.

### Accession numbers

Accession number of sequences on *M*. *ulcerans* gene:

- *IS2404* (GenBank accession no. BX649209).
- *IS2606* (GenBank accession no. BX649209).
- *KR-B* (GenBank accession no. BX649209).

## Results

Whatever the qPCR run, all the negative extraction control and the no template control, NTC did not show an amplification curve. This means the absence of any contamination during the extraction process and from the water used to prepare the master mix. All samples that did not generated an amplification curve were considered negative to the targeted sequences.

The internal control (IPC) and the positive control showed exponential amplification curves. All sample having an exponential curve like the positive control with the Ct<40, was considered as positive to the targeted sequence.

*M*. *ulcerans* genetic profile detected in environmental samples.

A total of 219 samples were analysed using real time PCR (qPCR) technique to determine *IS2404* and *IS2606* insertions and KR-B gene sequences.

for the *IS2404*-qPCR, 10 (5%) samples did not show an amplification curve neither for the internal control IPC nor the *IS2404* sequence indicating an inhibition reaction. After 10-fold dilution and retesting, these samples were negative for *IS2404* sequence. Overall, 37 (17%) out of 219 samples analysed were tested positive for *IS2404* insertion with Ct values ranging from 26.6 to 38.3, suggesting a probable presence of *M*. *ulcerans* in environmental samples (Table 2). The calibration curve generated by *IS2404*-qPCR results of plasmid standards has shown high detection sensitivity up to 0.01 copies of the *M*. *ulcerans* genome in a sample (S1 Table).

**Table 2:** Real-time PCR results of environmental samples tested positive by Ct values of *IS2404* and *IS2606* insertions and KR-B gene, Zio and Yoto districts, maritime region, Togo, May 19–30, 2015.

Site*	Ct (*IS2404*) n = 37	Ct (*IS2606*) n = 14	Ct (KTR) n = 6	ΔCt (*IS2404-IS2606*) n = 14	Profile n = 6
**FA1**	36.8	37.3		0.5	
**FA2**	**35.1**	**35.5**	**37.2**	0.4	***M*. *ulcerans***
**FA3**	37,3				
**FA4**	**36.8**	**38.4**	**38.2**	1.6	***M*. *ulcerans***
**FA5**	37,4				
**FA6**	36.5				
**FB1**	37.,4				
**FB2**	36.4	34.5		1.9	
**FB3**	36,5	36.6		0.1	
**FB4**	36.8			0.1	
**FZ1**	26.6				
**FZ2**	38.3				
**FZ3**	36.8	38.3		1.5	
**GA1**	34.9				
**GA2**	37.0	36.3		0.7	
**GA3**	35.9				
**GA4**	29.3				
**GA5**	31.6				
**GA6**	37.6				
**GKP1**	37.4	37.7		0.3	
**GKP2**	**35.6**	**36,4**	**36.4**	0.8	***M*. *ulcerans***
**KZ1**	35.3				
**YS1**	35.7	37.5		1.8	
**YS2**	34.9				
**YS3**	36.4	33.7		2.7	
**YS4**	26.9				
**YS5**	**35.7**	**36.4**	**36.2**	0.7	***M*. *ulcerans***
**YS6**	37.4				
**TDV1**	31.9				
**TDV2**	29.8				
**TDV3**	**35.8**	**37.4**	**37.7**	1.6	***M*. *ulcerans***
**TDV4**	27.0				
**TDV5**	31.9				
**TA1**	28.3				
**TA2**	37.1				
**TA3**	33.5				
**TDD1**	**33.6**	**35.5**	**36.5**	1.9	***M*. *ulcerans***

*Site describes villages where samples were collected with numerical number indicated the different points of collection; Ct: Cycle threshold; ΔCt: Mean of the difference between IS*2404* and IS2606

To confirm the presence of *M*. *ulcerans* in environmental samples, all the above mentioned *IS2404* positive samples were also tested for *IS2606* insertion sequence and KR-B gene. Thus, out of 37 *IS2404*-positive samples, 14 (38%) samples were tested positive for *IS2606* with Ct values varying from 33.3 to 38.3. The difference (ΔCt) between *IS2404* and *IS2606* (*IS2606-IS2404*) was analysed and presented in Table 2. The mean Ct difference between *IS2404 and IS2606* was 1.1 (interval from 0.3 to 1.9) (Table 2). This ΔCt value was < 7 indicating that DNA amplified belongs to *M*. *ulcerans strains*, lineage 3 which are found in human lesions and contain high copies of *IS2606* per genome and not for other mycobacteria or non-virulent mycobacteria (lineage 1) which are fish and frog pathogens, or lineage 2 *M*. *ulcerans*, both of which harbor only few copies of *IS2606* [19–21].

Finally, 6 (43%) out of 14 samples that containing both *IS2404* and *IS2606* insertions sequences were tested positive for mycolactone KR-B gene. In conclusion, we detected 6 (2.7%) samples positive for *M*. *ulcerans* (*IS2404/IS2606/KR*) out of 219 analysed (Table 2).

### Identification of environmental reservoirs for *M*. *ulcerans*

To identify possible reservoirs of *M*. *ulcerans* in the environment of BU patients, samples from different sources were tested and consisted of water (n = 65), plants (n = 29), soil (n = 119) and animal faeces (n = 6) (Table 3). The distribution of the number of samples tested overall and tested positive according the source is presented in Table 3.

**Table 3:** Distribution of number of samples tested overall and number of tested positive by different matrices, Zio and Yoto districts, maritime region, Togo, May 19–30, 2015.

Environnemental samples matrices	Number *IS2404* positive/Number of samples tested (%)	Number *IS2606* positive/Number of *IS2404* positive (%)	Number KR-B positive/Number *IS2606* positive (%)	*M*. *ulcerans/* number of samples tested (%)
**Water (n = 65)**	**10/65 (15.4)**	**4/10 (40.0)**	**1/4 (25.0)**	**1/65 (1.5)**
Stagnant water (n = 14)	5/14 (35.7)	2/5 (40.0)	0/2 (0.0)	0 (0.0)
Open borehole/cistern water (n = 10)	1/10 (10.0)	0/1 (0.0)	NA	0 (0.0)
River water (n = 30)	4/30 (13.3)	2/4 (50.0)	1/2 (50.0)	1 (3.3)
pump/borehole water (n = 11)	0/11 (0.0)	NA	NA	0 (0.0)
**Vegetal flora (n = 29)**	**6/29 (17.2)**	**3/6 (50.0)**	**3/3 (100.0)**	**3/29 (10.3)**
Plants along river (n = 20)	5/20 (25.0)	2/5 (40.0)	2/2 (100.0)	2/20 (10.0)
Plants around stagnant water (n = 9)	0/9 (0.0)	NA	NA	0 (0.0)
**Soil (n = 119)**	**20/119 (16.8)**	**7/20 (35.0)**	**2/7 (28.6)**	**2/119 (1.7)**
Mud along river (n = 25)	8/25 (32.0)	3/8 (37.5)	1/3 (33.3)	1/25 (4.0)
Mud around stagnant water (n = 20)	2/20 (10.0)	0/2 (0.0)	NA	0 (0.0)
Soil from houses and other (n = 74)	10/74 (13.5)	4/10 (40.0)	1/4 (25.0)	1/74 (1.3)
**Animal feces (n = 6)**	**1/6 (16.7)**	**0/1 (0.0)**	**NA**	**0 (0.0)**
**Total (n = 219)**	**37/219 (16.9)**	**14/37 (37.8)**	**6/14 (42.8)**	**6/219 (2.7)**

KR-B: Ketoreductase-B domain

Overall, *IS2404* positivity rate was not significantly different (p = 0.79) between water source (15%), plants (17%), soil (17%) and animals’ feces (17%) (Table 3). For *IS2606* sequence, a high positivity rate was observed for water (40%) and biofilms/plants (50%) but were not significantly different from soil samples (35%; p = 0.84) (Table 3). The KR gene was found at 100% in the biofilm/plants samples compared to water (25%) and soil (29%). However, there was no significant difference of KR-B proportion between these three matrices (p = 0.99) (Table 3).

In Table 3, we observed that 1/10 water sample from open borehole/cisterns was tested positive for *IS2404* but did not reveal amplification of *IS2606* and KR gene sequences. From water of pump/borehole, no sample was tested positive for both insertions sequences and the KR gene. Stagnant water samples (5/14) and water from rivers (4/30) were tested positive for *IS2404*. The two matrices had high positivity rates for IS*2606* (≥40%). However, the KR-B gene was only detected in water samples (50%) collected form rivers (Table 3).

Concerning biofilms/plants, samples collected from ponds did show amplification for the three sequences. However, samples collected from rivers, at the surface and at along edges were positive for *IS2404* in 25% (5/20) and *IS2606* in 40% (2/5) insertions sequences. The positivity rate of KR-B gene was found in the 2 samples (100%) tested *IS2606*-positive (Table 3).

For soil samples, *IS2404* insertion was positive in samples collected along river banks (8/20), around stagnant water (2/20) and in homes and other locations (10/74). However, *IS2606* sequence was detected in 37% (3/8) of samples collected along river banks and in samples from homes and other locations in 40% (4/10). The KR-B gene was detected in these two sources in more than 25% (Table 3).

Only one sample of animal feces was tested positive for *IS2404* but negative for other sequences (*IS2606*/KR-B gene) (Table 3).

In summary, *M*. *ulcerans* including three sequences (*IS2404/IS2606/KR*) were detected in matrices consisting of water in 1.5% (1/65); biofilms/plants in 10.3% (3/29) and soil in 1.7% (2/119) (Table 3). However, there was no significant predominance (p = 0.96) between all sources of matrixes tested.

### Comparison of *M*. *ulcerans* genetic profiles between environmental and clinical samples

This comparison aims to establish a link between the disease and *M*. *ulcerans* in the living milieu of Buruli ulcer patients. Then, 50 clinical samples of BU patients have been tested using qPCR in the same conditions as describe for environmental samples. The results of qPCR for clinical samples with comparison with environmental samples are presented in Table 4. The real time PCR had showed that all DNA tested from clinical samples was positive for both the two insertions (*IS2404/IS2606*) and the KR-B gene sequences (Table 4).

**Table 4:** Comparison of *M*. *ulcerans* profiles detected in clinical and environmental samples by Ct value of *IS2404/IS2606* and KR-B sequences detected, districts of Zio and Yoto, maritime region, Togo, May 19–30, 2015.

Sample types	Ct (*IS2404*)	Ct (*IS2606*)	Ct (KTR)	ΔCt (*IS2606-IS2404*)	Profile
**Environnemental samples (n = 6)**					
Vegetal flora (n = 3)	**35.83**	**36.77**	**37.27**	0.94	***M*. *ulcerans***
Soil (n = 2)	**34.65**	**35.95**	**36.35**	1.30	***M*. *ulcerans***
River water (n = 1)	**35.80**	**37.40**	**37.70**	0.30	***M*. *ulcerans***
**Clinical samples (n = 50)**					
Fine needle liquid aspiration (FNA; n = 31)	**24.36**	**24.72**	**27.51**	0.36	***M*. *ulcerans***
Swabs (n = 19)	**25.12**	**26.32**	**30.00**	1.20	***M*. *ulcerans***

Ct: Cycle threshold; ΔCt: Mean of difference between *IS2606 and IS2404*

The analysis of the mean ΔCt of the difference between *IS2404* and *IS2606* had showed that the ΔCt was < 7 for the swabs and FNA (Table 4). This mean ΔCt confirms that DNA detected in clinical samples is belonging to *M*. *ulcerans* and not for any other mycolactone- producing mycobacteria [19, 21]. The comparison of this result obtained in clinical samples (ΔCt < 7) to the one found in environmental samples had led to conclude that the *M*. *ulcerans* could have the same genetic profile in the two types of samples.

**Fig 2:**
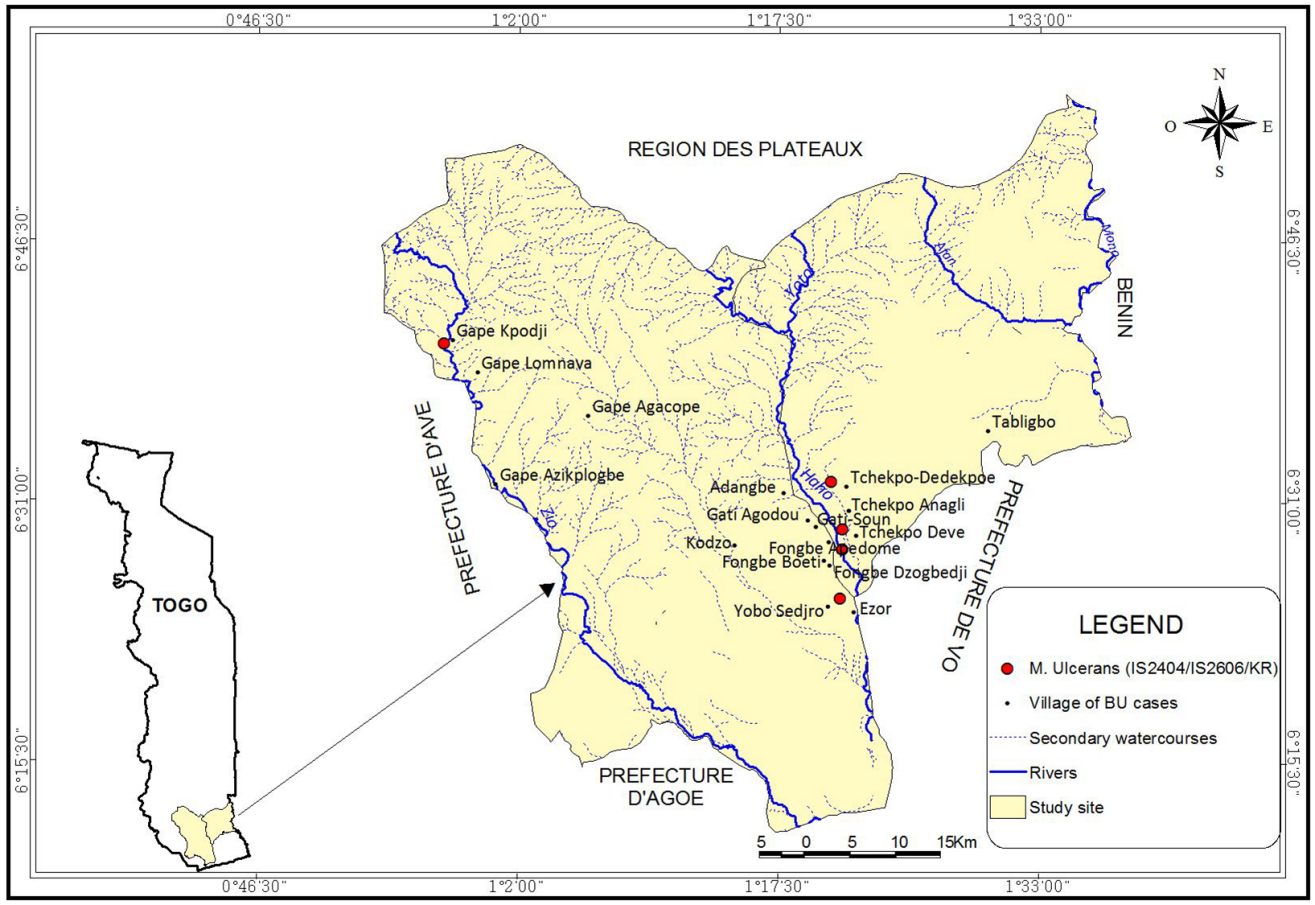
Geographical distribution of *M*. *ulcerans* profiles detected in the environment of districts of Zio and Yoto, maritime region, Togo May 19–30, 2015. The circles in red correspond to the *M*. *ulcerans* strains with three sequences (*IS2404/IS2606*/KR). This profile is found in the villages around the Haho river with some cases at the neighbourhood of Zio river.

The *M*. *ulcerans* detected had a distribution limited to 4 villages including Fongbé Apédomé (2 cases), Yobo Sedjro (1 case), Tchékpo Dévé (1 case) and Tchékpo Dedekpoe (1 case) around the Haho river. Only one case was detected in the village of Gapé Kpodji near the Zio River (Fig 2). This distribution of environmental samples is like the confirmed Buruli ulcer patients in the same villages of residence (Fig 1). However, there was no significant correlation (p = 0.59) between the number of BU patients and the presence of *M*. *ulcerans* in the environment.

## Discussion

Based on epidemiological evidences [12–13, 21–24], it has been suggested that *M*. *ulcerans* is an environmental organism that sometimes infects humans [25]. The mode of transmission is still poorly understood although primary contact of the skin with contaminated aquatic environment is a possible route of infection [25]. In order to improve the understanding of the mode of transmission of *M*. *ulcerans* in human, it is important at first stage to determine the ecology of the *M*. *ulcerans* [39]. For this purpose, this study aimed to determine the presence of *M*. *ulcerans* in the environment and its relationship with BU disease.

The real time PCR analysis of environmental samples in our study has shown that *M*. *ulcerans* including three sequences (*IS2404/IS2606*/KR) was detected in 2.7% (6/219) of samples tested. This is the first evidence that *M*. *ulcerans* is present in the environment of the Zio and Yoto districts of the maritime region in Togo. This percentage is higher than what was found in Ghana [40]. However this frequency remained lower than the positivity rate found in other studies conducted in Africa [12,22–23,29], South America [39] and Australia [13]. The difference between frequencies observed may be explained by the criteria of *M*. *ulcerans* detection. In our study, the confirmation method of the presence of the *M*. *ulcerans* was based on the detection of three sequences including two insertions (*IS2404 and IS2606*) and the mycolactone KR-B gene. This method has been used in Cameroon [12] and in Ghana [40]. However, in other studies from Benin [22], Côte d’Ivoire [29] and South America [39], the authors have detected two sequences consisting of *IS2404* insertion and KR-B gene. On the other hand, Stinear [13] had identified *M*. *ulcerans* in Australia, basing only on the two insertions (*IS2404/IS2606*) without detecting the KR-B gene.

Indeed, although *IS2404* sequence is considered a specific marker of *M*. *ulcerans* detection in clinical samples [19], the existence of *M*. *ulcerans* ecotypes (lineage 1) positive for this sequence which are largely non-virulent for humans complicates the interpretation of real-time PCR results in environmental samples [19, 21]. This requires that samples should be also tested for detection of *IS2606* sequence and the difference ΔCt between *IS2606* and *IS2404* will be analysed [21]. The *M*. *ulcerans* (lineage 3) ecotypes that cause human diseases in Africa and Australia incorporate a higher number of *IS2606* sequences than the lineage 1 [21]. Thus, such ecotypes can be differentiated based on the ΔCt value of the difference between *IS2606* to *IS2404*. Indeed, a ΔCt <7 allow to identify the ecotype of *M*. *ulcerans*, (subspecies) ulcerans, which are virulent strains of lineage 3 compared to other non-ulcerans mycolactone- producing mycobacteria (MPM) or non-*M*. *ulcerans* strains virulent (lineage 1) [19–21].

To determine probable reservoirs or habitats of *M*. *ulcerans*, we analysed environmental samples. The qPCR results showed that samples of biofilms/plants (10.3%), soil (1.7%) and river water (1.5%) were positive to *M*. *ulcerans* DNA including three sequences (*IS2404/IS2606/*KR). However, the positivity rate of *M*. *ulcerans* was not significantly different between these three sources. In contrast to this observation, other studies [12,22,29,39] had often identified *M*. *ulcerans* DNA in a large proportion of water samples. Whilst it could be concluded that mycobacteria are more frequent in water, the difference between these results may be due to the DNA extraction method and the sample collection site. In general, water samples are collected in a large volume with possibility of concentration on filters, whereas samples of biofilms/plants and soil are used in small quantities (0.25 g) according to the kits available for DNA extraction [39]. The site collection of samples could explain the prevalence of *M*. *ulcerans* DNA as some authors have especially collected samples from aquatic areas [13, 23, 39] which are at high risk of *M*. *ulcerans* infection while other performed sampling in both aquatic and dry areas [12, 22, 29].

Due to the abundance of other and faster growing microorganisms in the environment, routine cultivation of *M*. *ulcerans* from environmental samples has mostly failed [8–9]. Because of theses relative difficulties, the real-time PCR is commonly used to confirm the presence of *M*. *ulcerans* in the environment by amplifying DNA of the bacteria [21]. Although this method does not allow to prove the presence of viable mycobacteria in a sample, in the absence of a culture isolate, concurrent detection of *IS2404* and *IS2606*, can be used to provide convincing evidence of the presence of *M*. *ulcerans* [41].

The relationship between the presence of *M*. *ulcerans* in environment and Buruli ulcer disease was analysed. Thus, using qPCR to detect three markers in the *M*. *ulcerans* genome, we found that the genetic profile detected was similar between environment and clinical samples. In addition, this mycobacterium identified in environmental samples in our study had similar distribution to BU patients in the same villages where they resided (Fig 2). This suggests a co-existence between patients and the pathogen in the same environment. Also, Possible reservoirs identified in environmental samples were water, biofilms/plants and soil (mud) which was collected at the surface or the edges of Haho and Zio rivers. The environment of these rivers could be potential source *M*. *ulcerans* infection in human. This observation confirms results from other studies [15–16,22–23,26,29,39] that aquatic sources are important source of risk of contracting Buruli ulcer. Furthermore, our study identifies the presence of *M*. *ulcerans* in living houses of BU patients (1.3%; Table 3) in dry areas. This result could explain that this bacterium was widely distributed in both aquatic and dry zones.

This study was limited by the lack of culture isolates of *M*. *ulcerans* from environmental samples. This should provide an undeniable proof of the presence of the viable mycobacteria and its association with Buruli ulcer disease. In the other hand, the absence of data from non-endemic areas has reduced the knowledge about the real distribution of *M*. *ulcerans* in the environment in surveyed districts.

### Conclusion

This study confirms the presence of *M*. *ulcerans* in the environment of the districts of Zio and Yoto in the maritime region of south Togo. This may explain partially, the high rates of Buruli ulcer patients in this region. Possible reservoirs of *M*. *ulcerans* identified were water, biofilms/plants and soil in the neighbourhood of rivers. Haho and Zio rivers could be potential sources of *M*. *ulcerans* infection in Human in these districts of the maritime region of south Togo.

## Supporting information

S1 Table Calibration curve of *IS2404* obtained from recombinant plasmid of *M*. *ulcerans*. (DOCX)
