# Supplementary material for: Molecular detection of Mycobacterium ulcerans in the environment and its relationship with Buruli ulcer occurrence in Zio and Yoto districts of maritime region in Togo
Source: PLoS Negl Trop Dis. 2018 May 21;12(5):e0006455. doi: 10.1371/journal.pntd.0006455 (PMC5983864; doi:10.1371/journal.pntd.0006455)
Supplement: S1 Table — (DOCX) [file pntd.0006455.s001.docx]

**Table S1**: Detection Limit of *IS2404*-qPCR based on *M. ulcerans* plasmid DNA

| **Series dilutions of *M. ulcerans* Plasmid DNA**  **(Copies/µl)** | **Ct (*IS2404*)** |
| --- | --- |
| 10^8^ | 13.0 |
| 10^7^ | 13.8 |
| 10^6^ | 14.9 |
| 10^5^ | 16.5 |
| 10^4^ | 21.1 |
| 10^3^ | 25.9 |
| 10^2^ | 28.2 |
| 10^1^ | 31.7 |
| 10^0^ | 34.3 |
| 10^-1^ | 36.0 |
| 10^-2^ | 38.0 |

Ct : cycle threshold
